# Aphthæ, or Vesicular Stomatitis of the Horse

**Published:** 1898-07

**Authors:** D. Hutcheon

**Affiliations:** Colonial Veterinary Surgeon


### SELECTIONS.

---

### APHTHÆ, OR VESICULAR STOMATITIS OF THE HORSE.

By D. Hutcheon,
COLONIAL VETERINARY SURGEON.

I observe that this disease is reported to have reappeared in the Transkeian Territories and some eastern districts.

*Nature of the Disease.* This is an eruptive febrile affection of the horse, characterized by the appearance of vesicles on the tongue, inside of the lips and cheeks, and frequently on the outside around the muzzle and nostrils. These vesicles—aphthæ—are irregular both in size and shape; but they are usually circular, of a yellowish color, and slightly raised above the surface of the mucous membrane. These vesicles burst and form ulcerous-looking sores, which often run together, leaving the tongue and lining of the lips and cheeks quite raw and very painful, rendering the animal unable to feed except with great difficulty. This last condition is most frequently observed in aged or debilitated horses. In a healthy, well-nourished animal the disease is generally mild, and invariably yields to simple treatment.

This disease is communicable by inoculation, but it does not appear to spread rapidly by infection. For instance, a healthy horse with a clean and uninjured mouth may eat out of the same manger with a horse which is suffering from the disease, and remain free from any appearance of it. But if there are any sores or abrasions about the horse's mouth, produced either by the bit or from feeding on thorny bushes, such a horse is very liable to become affected.

When a human being becomes inoculated through having cuts or abrasions of the skin or hands, very intractable sores are sometimes produced.

The disease appeared in the colony about the beginning of March, 1884, and was first reported to me from King Williamstown and Peddie districts. I was unable to find out where it originated. Previously to that it must have been very rarely met with, as many old and experienced farmers and horseowners informed me that they had not seen it before. Many were of the opinion that the disease was introduced by the horse eating prickly pears and thorny bushes, the ordinary food being very scarce at that time on account of a prolonged drought. But I saw it among horses in localities where there were no prickly pears on the veld, and also among stable-fed horses. It certainly prevailed most, and was most severe and intractable, amongst poor and debilitated horses, whether the poverty was induced by insufficient food or hard work.

Disorders of the digestive organs are recognized as a cause of this complaint, both by human and veterinary pathologists. It is quite possible, therefore, that the disease arose, in the first instance, from some peculiar local conditions, associated with the food or water, acting on an enfeebled constitution, but there can be little doubt that it assumed a contagious character of the nature above described.

The disease prevailed most along certain post-cart routes and among the horses belonging to the Cape police. When it entered a stable of post-cart horses it generally affected the majority of them. On the other hand, I know of numerous instances in private stables where one horse only was affected; although no efforts were made to isolate the sick one, or prevent him from eating and drinking out of the same vessels with the others, it disappeared very much as it came, without any very apparent reason. Since that time, however, frequent individual outbreaks of this disease have been reported; one of these occurred in the Swellendam district during the year 1895.

*Treatment.* This resolves itself into constitutional and local remedies.

First, with respect to constitutional treatment. If the affected animal is otherwise in good condition, give him a pint of raw linseed-oil, followed by a teaspoonful each of saltpetre and flowers-of-sulphur, daily, while the complaint lasts. If, on the other hand, the horse is poor and debilitated, add a teaspoonful of sulphate o iron to the above mixture, and supply him with soft, succulent, nourishing food, such as bran-mashes, green food, carrots, etc.

Second, local treatment. This consists in cleaning out the mouth three times a day with antiseptic astringent lotions, such as strong solutions of alum, bluestone, or boric acid, an ounce of either dissolved in a bottle of water. When the mouth is very sore, raw, and smells very badly, add half an ounce of carbolic acid, or an ounce of Jeyes' fluid to any of these mixtures. There are many other lotions which may be used, such as solutions of chlorate of potash, salicylic acid, and many owners use little else but common salt and sulphur placed in the mouth.

The simplest and easiest method of dressing a horse's mouth is to elevate the animal's head, then quietly introduce the neck of the bottle containing the lotion into the side of the mouth, and allow the mixture to run out of the bottle until the horse's mouth is pretty full, then gradually lower his head, when he will rinse out his mouth by the action of the tongue as the fluid trickles out. Do not attempt to throw or dash anything into a horse's mouth, or it will cause him to resist any subsequent dressing.—*Cape of Good Hope Agricultural Journal*, May, 1898.

